# Association Study between the *CD157*/*BST1* Gene and Autism Spectrum Disorders in a Japanese Population

**DOI:** 10.3390/brainsci5020188

**Published:** 2015-05-20

**Authors:** Shigeru Yokoyama, Naila Al Mahmuda, Toshio Munesue, Kenshi Hayashi, Kunimasa Yagi, Masakazu Yamagishi, Haruhiro Higashida

**Affiliations:** 1 Research Center for Child Mental Development, Kanazawa University, Kanazawa 920-8640, Japan; E-Mails: naila@med.kanazawa-u.ac.jp (N.A.M.); munesue@med.kanazawa-u.ac.jp (T.M.); haruhiro@med.kanazawa-u.ac.jp (H.H.); 2 MEXT Strategic Research Program for Brain Sciences (SRPBS), Okazaki 444-0840, Japan; 3 Division of Cardiovascular Medicine, Kanazawa University Graduate School of Medical Science, Kanazawa 920-8641, Japan; E-Mails: kenshi@med.kanazawa-u.ac.jp (K.H.); myamagi@med.kanazawa-u.ac.jp (M.Y.); 4 Medical Education Research Center, Kanazawa University Graduate School of Medical Science, Kanazawa 920-8640, Japan; E-Mail: diabe@med.kanazawa-u.ac.jp

**Keywords:** autism spectrum disorder, BST-1, CD157, single-nucleotide polymorphism

## Abstract

CD157, also referred to as bone marrow stromal cell antigen-1 (BST-1), is a glycosylphosphatidylinositol-anchored molecule that promotes pre-B-cell growth. Previous studies have reported associations between single-nucleotide polymorphisms (SNPs) of the *CD157/BST1* gene with Parkinson’s disease. In an attempt to determine whether SNPs or haplotypes in the *CD157/BST1* are associated with other brain disorders, we performed a case-control study including 147 autism spectrum disorder (ASD) patients at Kanazawa University Hospital in Japan and 150 unselected Japanese volunteers by the sequence-specific primer-polymerase chain reaction method combined with fluorescence correlation spectroscopy. Of 93 SNPs examined, two SNPs showed significantly higher allele frequencies in cases with ASDs than in unaffected controls (rs4301112, OR = 6.4, 95% CI = 1.9 to 22, *p* = 0.0007; and rs28532698, OR = 6.2, 95% CI = 1.8 to 21, *p* = 0.0012; Fisher’s exact test; *p* < 0.002 was considered significant after multiple testing correction). In addition, CT genotype in rs10001565 was more frequently observed in the ASD group than in the control group (OR = 15, 95% CI = 2.0 to 117, *p* = 0.0007; Fisher’s exact test). The present data indicate that genetic variation of the *CD157/BST1* gene might confer susceptibility to ASDs.

## 1. Introduction

Autism spectrum disorder (ASD) is a neurodevelopmental disorder characterized by social impairments, communication difficulties, and restricted repetitive behaviors [1]. As an animal model of ASD, we previously reported a mouse lacking *CD38* gene, which encodes a transmembrane protein with ADP-ribosyl cyclase activity [2]. In this mouse, which exhibits impaired social behaviors such as social amnesia and neglect-like maternal behavior, we demonstrated that decreased formation of cyclic ADP-ribose (cADPR) results in dysfunctional calcium (Ca^2+^)-induced Ca^2+^-release for the secretion of oxytocin (OXT), a neurohypophyseal hormone essential for social recognition [2]. Since then, there have been reported associations between single-nucleotide polymorphisms (SNPs) in the human *CD38* gene with ASDs [3,4]. The SNP rs3796863 (C > A) of the *CD38* gene was associated with high-functioning autism (HFA) in both samples from the Autism Gene Resource Exchange [3] and those from low-functioning autism subjects in Israel [4], but not in Japanese HFA subjects [3]. Another SNP rs1800561 (g.4693C > T; p.R140W) was found in three probands out of 29 ASD patients and in 10 family members of three pedigrees with variable levels of ASD or ASD traits [3]. The plasma levels of OXT in ASD subjects bearing the R140W variant were lower than those in ASD subjects homozygous for the major R140 allele [3].

CD157, also referred to as bone marrow stromal antigen-1 (BST-1), belongs to the NADase/ADP-ribosyl cyclase family, which also includes CD38 [5,6,7,8,9,10,11,12,13]. These two molecules share 36% of their amino-acid sequence, and serve as both a nicotinamide adenine dinucleotide (NAD)-metabolizing ectoenzyme and a signaling molecule whose role in polarization, migration, and diapedesis of human granulocytes has been documented. CD157/BST-1 was initially isolated as a cell-surface molecule that promotes the pre-B lymphocyte growth [8,14]. *CD157/BST1* plays a variety of roles in humoral immune responses, neutrophil transmigration and hematopoietic stem cell support [8,10,15,16,17]. CD157/BST-1 is also involved in the pathogenesis of various diseases, such as the survival of B lymphocytes in rheumatoid arthritis, the progression of leukemia, and metastasis of human ovarian carcinoma cells [8,13,18,19,20].

Recent genetic analysis identified the *CD157/BST1* gene on human chromosome 4p15 as a susceptibility marker for Parkinson’s disease [21,22,23,24,25,26,27,28,29,30]. Ceroni *et al.* reported a patient with autism and asthma whose *CD38* and *CD157/BST1* genes were partially deleted [31]. In addition, we have recently demonstrated that mice deficient in the *CD157/BST1* gene exhibited anxiety-related and depression-like behaviors [32]. It is unknown, however, whether variation of the *CD157/BST1* gene is associated with other brain disorders. In this study, therefore, we performed a case-control study to test *CD157/BST1* genetic variation for association with ASDs.

## 2. Experimental Section

### 2.1. Subjects

We recruited 147 ASD subjects (113 males, 34 females; 15.6 ± 0.6 years) from the outpatient psychiatry department of the Kanazawa University Hospital as previously described [3,33]. All subjects fulfilled the DSM-IV criteria for pervasive developmental disorder. The diagnoses were made by two experienced child psychiatrists through interviews and clinical record reviews, as described previously [3], and the subjects had no apparent physical anomalies. The two experienced child psychiatrists independently confirmed the diagnosis of ASD for all patients by semi-structured behavior observations and interviews with the subjects and their parents. At the interviews with the parents, which were helpful in the evaluation of autism-specific behaviors and symptoms, the examiner used one of the following methods: the Asperger Syndrome Diagnostic Interview [34], Autism Diagnostic Interview-Revised (ADI-R) [35], Pervasive Developmental Disorders Autism Society Japan Rating Scale [36], Diagnostic Interview for Social and Communication Disorders [37], or Tokyo Autistic Behavior Scale [38]. The 150 controls (115 males, 35 females; 23.8 ± 0.3 years) were unselected Japanese volunteers. All patients and controls were Japanese with no non-Japanese parents or grandparents. This study was approved by the ethics committees of Kanazawa University School of Medicine. All examinations were performed after informed consent according to the Declaration of Helsinki.

### 2.2. Genotyping

Genomic DNA was extracted as previously described [33] from venous blood samples using a kit (Wizard Genomic DNA Purification kit; Promega, Madison, WI, USA), or from nails using the (ISOHAIR DNA extraction kit; Nippon Gene, Tokyo, Japan). In some instances, genomic DNA samples were subjected to the whole-genome amplification method (the REPLI-g kit; Qiagen, Hilden, Germany). Then SNPs were determined at Kurabo Industries Ltd. (Osaka, Japan) by the sequence-specific primer (SSP)-PCR method combined with fluorescence correlation spectroscopy as described by Nishida *et al.* [39]. The SNPs selected for genotyping were mostly with a minor allele frequency (MAF) >0.1, as indicated by the dbSNP database [40], HapMap genome browser (release 27) [41], and 1000 Genomes Project database [42,43] in the JPT (Japanese in Tokyo, Japan), CHB (Han Chinese in Beijing, China) plus JPT, and global populations (Supplementary Table S1). These SNPs were located in a region covering the *CD157/BST1* gene (chr4:15704573–15733796, based on the human genome assembly GRCh37/hg19 at the UCSC Genome Bioinformatics Site [44]. The most upstream and downstream SNPs were rs112044965 at chr4:15704603 and rs11934811 at chr4:15738253, respectively. Inter-SNP distance was less than 2 kb.

Linkage disequilibrium (LD) blocks in our sample were analyzed by HaploView 4.2 [45].

### 2.3. Statistical Analysis

Genotype and allele frequencies were analyzed using a contingency table and the Fisher exact test (GraphPad Prism 6; GraphPad Software Inc., San Diego, CA, USA), and *p*-values smaller than 0.05 were considered to be statistically significant. Multiple-testing correction was performed after controlling for LD between the selected SNPs by the method of Nyholt [46,47]. The estimated effective number for independent loci was 23 and α was estimated to be equal to 0.002. *p*-Values below 0.002 were thus considered significant for single SNP association analysis.

Hardy-Weinberg Equilibrium (HWE) was tested by both Pearson’s chi-square goodness-of-fit test [48] and likelihood ratio test [49].

Statistical power was calculated using the Genetic Power Calculator [50,51]; calculations were undertaken assuming a population prevalence of 0.015 for ASD [52], a false-positive rate (α) of 0.05, and a *D′* value of 1 between the marker and disease, with a false positive rate of 5%. Alternatively, Chi-squared power calculation was done using the statistical package R; effect sizes were calculated following the method described by Chinn [53].

## 3. Results

**Figure 1:**
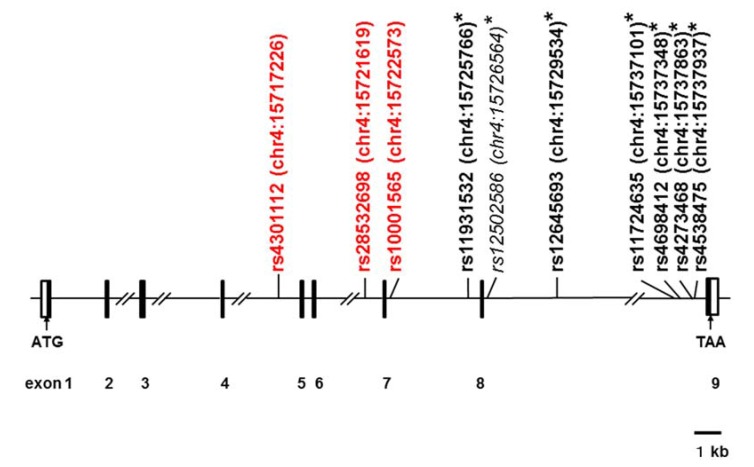
Schematic genomic structure of the human *CD157/BST1* gene and locations of single-nucleotide-polymorphisms (SNPs). The exon-intron organization is depicted based on GenBank accession numbers NM_004334 and NC_000004. Black and open boxes represent protein-coding regions and untranslated regions, respectively. The SNPs (indicated in bold type) are selected from those statistically analyzed in this study; rs12502586 was not tested (plain and italicized). Red lettering represents SNPs that showed significant association with ASD in allele and/or genotype frequencies in the present study; asterisks indicate those previously reported as Parkinson’s disease-associated markers [21,23,24,25,27,28,29,30]. The locations of the SNPs on human chromosome 4 (chr4) are indicated in parentheses; numbers after colons represent genomic positions based on the human genome assembly GRCh37/hg19 at the UCSC Genome Bioinformatics Site [44].

Of 121 SNPs examined (Supplementary Table S1), 93 with a high success rate (>95%) were further subjected to statistical analysis. Among them, three SNPs showed significantly higher allele frequencies in cases with ASDs than in unaffected controls (rs4301112, OR = 6.4, 95% CI = 1.9 to 22, *p* = 0.0007; rs28532698, OR = 6.2, 95% CI = 1.8 to 21, *p* = 0.0012 and rs10001565, OR = 5.5, 95% CI = 1.6 to 19, *p* = 0.0038; Fisher’s exact test; Table 1). rs4301112, rs28532698, and rs10001565 are located in introns 4, 6, and 7, respectively (Figure 1). After multiple testing correction for effective total number of SNPs, significantly higher allele frequency was observed in rs4301112 and rs28532698, but not in rs10001565 (Table 1). In rs10001565, only C/T genotype was significantly more frequent in the ASD group than in the unaffected control group (OR = 15, 95% CI = 2.0 to 117, *p* = 0.0007; Fisher’s exact test; Table 1).

The SNPs analyzed in this study included the previously reported Parkinson’s disease-associated ones: rs11931532 [21,23]; rs12645693 [21,23]; rs4698412 [21,23,24]; rs4538475 [21,23,27]; rs11724635 [27,28,30]; rs12502586 [25]; and rs4273468 [29] (Figure 1). However, these SNPs did not show significant association with ASD at all (Supplementary Table S2).

We then analyzed the data based on three different genetic models. The three SNPs (rs4301112, rs28532698, and rs10001565) showed significant associations with ASD in a recessive model, but not in additive and dominant models (Supplementary Table S3). Recessive model exhibited low *p*-values (rs4301112, OR = 8.9, 95% CI = 2.0 to 39.6, *p* = 0.0005; rs28532698, OR = 8.7, 95% CI = 2.0 to 38.8, *p* = 0.0009 and rs10001565, OR = 8.1, 95% CI = 1.8 to 36.3, *p* = 0.0038; Fisher’s exact test; Supplementary Table S3).

Deviations from HWE in the control group were observed in the three SNPs (*p* = 1.11E−15 for rs4301112, *p* = 2.19E−15 for both rs28532698 and rs10001565; Fisher’s exact test; Supplementary Table S4). In addition, we assessed HWE in three genetic models by the likelihood ratio test [49]. Higher *p*-values (>0.05) were obtained in the recessive model (*p* = 0.0870 for rs4301112, *p* = 0.0876 for rs28532698, and *p* = 0.0993 for rs10001565; Supplementary Tables S4–S6).

Using the Genetic Power Calculator [50,51], the power of a significance test (type I error rate of 0.05, Table 1) was calculated to be 1.0 for the three SNPs. In the three genetic model analysis; the highest statistical power was 1.0 under a recessive model for the three SNPs, with lowest value being 0.05 for rs1001565 under a dominant model (Supplementary Table S3).

**Table 1:** Comparison of genotype and allele frequencies of rs4301112, rs28532698 and rs10001565 at Kanazawa University Hospital for autism spectrum disorders (ASDs).

	Cases	Control	Odds Ratio	*p*
			(95% CI)	
rs4301112				
Genotype	(*n* = 145)	(*n* = 146)		
A/A	129 (88.9%)	144 (98.6%)	Referent	
A/G	14 (9.7%)	1 (0.7%)	16 (2.0, 121)	***0.0004***
G/G	2 (1.4%)	1 (0.7%)	2.2 (0.20, 25)	0.6054
Allele	(*n* = 290)	(*n* = 292)		
A	272 (93.8%)	289 (99%)	Referent	
G	18 (6.2%)	3 (1.0%)	6.4 (1.9, 22)	***0.0007***
rs28532698				
Genotype	(*n* = 145)	(*n* = 143)		
A/A	129 (88.9%)	141 (98.6%)	Referent	
A/G	14 (9.7%)	1 (0.7%)	15 (2.0, 118)	***0.0007***
G/G	2 (1.4%)	1 (0.7%)	2.2 (0.20, 24)	0.6090
Allele	(*n* = 290)	(*n* = 286)		
A	272 (93.8%)	283 (99.0%)	Referent	
G	18 (6.2%)	3 (1.0%)	6.2 (1.8, 21)	***0.0012***
rs10001565				
Genotype	(*n* = 145)	(*n* = 143)		
C/C	130 (89.7%)	141 (98.6%)	Referent	
C/T	14 (9.7%)	1 (0.7%)	15 (2.0, 117)	***0.0007***
T/T	1 (0.7%)	1 (0.7%)	1.1 (0.067, 18)	1.0000
Allele	(*n* = 290)	(*n* = 286)		
C	274 (94.5%)	283 (99%)	Referent	
T	16 (5.5%)	3 (1%)	5.5 (1.6, 19)	0.0038

CI, confidence interval; *p*-Values obtained by Fisher’s exact test are given; Significant *p*-values after multiple testing correction for effective total number of SNPs are written in bold and italicized.

Haplotype analysis revealed that 13 cases (9.0%, *n* = 145) carried all the minor alleles of the three SNPs (AG/AG/CT for rs4301112-rs28532698-rs10001565), whereas only one (0.7%, *n* = 141) did in the control group (OR = 14.2, 95% CI = 1.4 to 110; Table 2).

**Table 2:** Haplotype frequencies of the *CD157/BST1* gene.

Haplotype Combination *	Cases	Control	Odds Ratio	*p*
	(*n* = 145)	(*n* = 146)	(95% CI)	
AA/AA/CC	129 (89.0%)	141 (96.6%)	Referent	
AG/AG/CT	13 (9.0%)	1 (0.7%)	14.2 (1.8, 110)	0.0014
GG/GG/TT	1 (0.7%)	1 (0.7%)	1.1 (0.1, 17.7)	1.000
AG/AG/CC	1 (0.7%)	0 (0.0%)	3.3 ** (0.1, 81.3)	0.480
GG/GG/CT	1 (0.7%)	0 (0.0%)	3.3 ** (0.1, 81.3)	0.480
Other types	0 (0.0%)	0 (0.0%)	-	
Undetermined	0 (0.0%)	3 (2.1%)	-	

CI, confidence interval; *p-*Values obtained by Fisher’s exact test are given; * Order of polymorphisms for the haplotypes is as follows: rs4301112, rs28532698 and rs10001565; ** Odds Ratios were calculated by adding 0.5 to each value.

**Figure 2:**
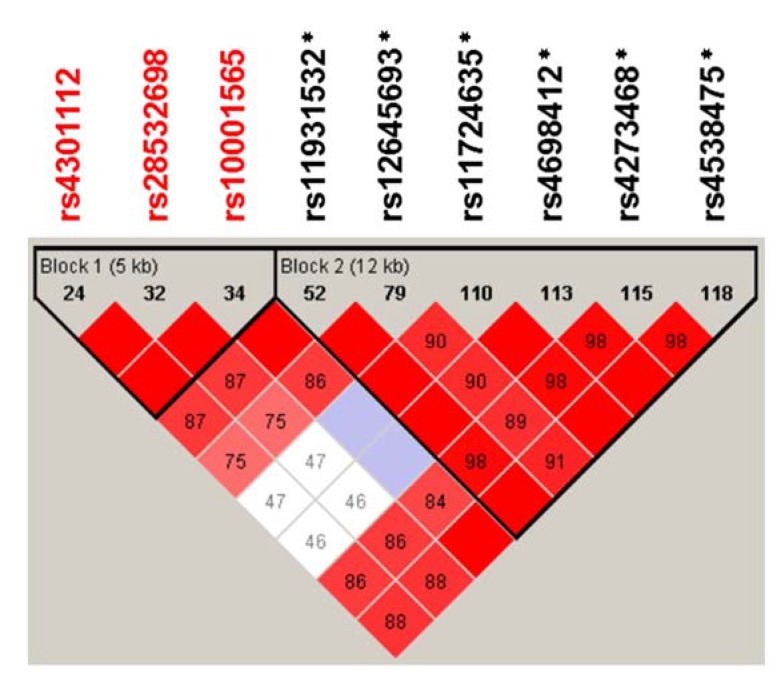
Linkage disequilibrium plot of the *CD157/BST1* gene in the sample studied. Numbers in the squares indicate *D′* values. Rs numbers in *red* represent SNPs that showed significant association with ASD in this study; asterisks denote those previously reported as Parkinson’s disease-associated markers [21,23,24,25,27,28,29,30].

LD analysis of these SNPs identified two haplotype blocks: a 5-kb one comprising the ASD-associated rs4301112, rs28532698 and rs10001565 (Block 1; Figure 2), and a 12-kb one including the SNPs associated with Parkinson’s disease (Block 2; Figure 2).

## 4. Discussion

In this study, we performed a case-control study in a Japanese population to test for genetic association between 93 SNPs in the *CD157/BST1* gene and ASDs. Our results show three possible risk SNPs for ASDs. As these SNPs are in high LD, it is likely that the results represent only one effect.

Functional annotation using HaploReg [54,55] suggests that genetic variations at rs4301112, rs28532698, and rs10001565 could alter binding sites for neural development-related transcription factors, histone deacetylase C2 (HDAC2 [56]), POU class 6 homeobox 1 (POU6F1 [57]), and HES-related family bHLH transcription factor with YRPW motif 1 (HEY1 [58]), respectively.

Additionally, in the UCSC (GRCh37/hg19) track “Transcription Factor ChIP-seq (161 factors) from ENCODE [59,60] with Factorbook Motifs”, the LD block 1 between rs4301112 and rs10001565 (Chr4: 15717226–15722573) includes predicted binding sites for c-Jun, STAT3 (signal transducer and activator of transcription 3), FOXP2 (forkhead box protein P2), PolR2a (Polκ RNA polymerase II polypeptide A), Elf-1 (E74-like factor 1), HNF4G (hepatocyte nuclear factor 4 gamma), HNF4A (hepatocyte nuclear factor 4 alpha), JunD, and C/EBPβ (CCAAT/enhancer binding protein beta). These sites are also overlapped with a peak of H3K27Ac Mark track, where acetylation of lysine 27 of the H3 histone protein is thought to enhance transcription [61] and possibly regulates brain development [61,62]. Of these transcription factors, FOXP2 is of particular interest, because its genetic abnormalities have been implicated in speech and language disorders [63,64]. A chromosomal translocation disrupting the *FOXP2* gene and a point mutation causing an amino-acid substitution in its forkhead domain have been identified in patients with severe developmental disorders of speech and language [63]. FOXP2 mRNA is expressed in the developing human brain, in good concordance with anomalous sites identified by brain imaging in adult speech and language disorders [64]. In this study, ASD-associated SNPs were located separately from Parkinson’s disease-associated ones. It is tempting to postulate that, during early brain development, CD157/BST-1 expression is under FOXP2-mediated transcriptional control, which may not involve the region containing Parkinson disease-associated SNPs. Future studies will be directed to explore these possibilities experimentally.

The limitation of this study is that sample size is small. In particular, the heterozygote numbers observed were small in both case and control groups, resulting in deviation from HWE and limited reliability and usefulness of the three SNPs as biomarkers. Although our results favor a recessive model, effect size of the *CD157/BST1* genetic variants should be carefully estimated. We tested HWE in unselected Japanese populations deposited in the HapMap [41], 1000 Genomes Project database [42], and human genome variation database [65], but did not detect any deviation in all seven available entries (one for rs4301112, one for rs28532698 and five for rs10001565; Supplementary Table S7). The reason for this discrepancy remains unknown: we have not recognized population stratification, admixture and cryptic relatedness among the subjects in this study. Future studies with larger sample size and/or family-based association testing are needed. Additionally, there are ethnic differences in allele frequencies; global MAFs for rs4301112, rs28532698 and rs10001565 are nearly 16%, whereas those in unselected Japanese populations are as low as 3% (the 1000 Genomes Project database [42,43], Supplementary Table S1). Therefore, replication in independent populations with various ethnic backgrounds is necessary.

## 5. Conclusions

We report association between SNPs (rs4301112, rs28532698, and rs10001565) located in the *CD157/BST1* gene with ASD. Our results warrant further analysis of *CD157/BST1* variants in ASD patients.

## Supplementary Files

Supplementary File 1
