# Supplementary material for: Association Study between the CD157/BST1 Gene and Autism Spectrum Disorders in a Japanese Population
Source: Brain Sci. 2015 May 20;5(2):188–200. doi: 10.3390/brainsci5020188 (PMC4493464; doi:10.3390/brainsci5020188)
Supplement: Supplementary File 1 [file brainsci-05-00188-s001.pdf]

Supplementary Information

Table S1. Single-nucleotide polymorphisms (SNPs) tested in the present study.

#	SNPs (dbSNP)	Minor Allele Frequencies			Success Rate (%)
		1000 Genomes *		HapMap **	
		Global	JPT	JPT	
1	rs112044965	0.015	0		67.4
2	rs2302468	0.076	0.178		0
3	rs2302467	0.493	0.399	0.41	0
4	rs11947383	0.407	0.279	0.28	78.7
5	rs3756247	0.246	0.077	0.10	96.3
6	rs3756246	0.422	0.308	0.29	95.3
7	rs76682013	0.091	0.173		97
8	rs55735476	0.059	0.053		97.3
9	rs2302466	0	0.005		96.7
10	rs73115627	0.133	0.173		96.3
11	rs28482082				0
12	rs35519415	0.499	0.462		0
13	rs4257653	0.228	0.077		96.3
14	rs4613560	0.113	0.048		97.3
15	rs4631042	0.474	0.457		85
16	rs4541502	0.491	0.466	0.48	96.3
17	rs55757331	0.058	0.048		97.7
18	rs16892260	0.263	0.457	0.39	96
19	rs73224659	0.151	0.231		95
20	rs111520831	0.004	0		98
21	rs116158357	0.007	0		97.7
22	rs73224660	0.151	0.231		95.3
23	rs73224662	0.17	0.236		98.3
24	rs4301112	0.164	0.029		97.7
25	rs3213710	0.443	0.466	0.47	97.3
26	rs4326008	0.113	0.048	0.05	98
27	rs6842052	0.216	0.231		95.7
28	rs73121382	0.13	0.183		96.3
29	rs75476202	0.007	0		98.3
30	rs10008644	0.331	0.26		97.3
31	rs12645405	0.428	0.486		96.7
32	rs28532698	0.164	0.029		96.7
33	rs7699180	0.12	0.053		96.7
34	rs10001565	0.163	0.029	0.04	96.7
35	rs9942275	0.17	0.245	0.24	97
36	rs9291637	0.351	0.332	0.28	96.7
37	rs10018756	0.271	0.303		96.7
38	rs9942211	0.123	0.25	0.23	97.3
39	rs9942247	0.174	0.255	0.22	95.3
40	rs73121395	0.172	0.25		97.3
41	rs35022883	0.349	0.332		99

Table S1. *Cont.*

#	SNPs (dbSNP)	Minor Allele Frequencies			Success Rate (%)
		1000 Genomes *		HapMap **	
		Global	JPT	JPT	
42	rs111796073	0.158	0.25		95.3
43	rs75219005	0.115	0.25		94.4
44	rs76579060	0.115	0.25		97.3
45	rs7688526	0.121	0.26	0.24	97
46	rs7672395	0.123	0.269	0.26	97.3
47	rs12642189	0.151	0.26		99.7
48	rs12651490	0.237	0.462		97.3
49	rs9999956	0.269	0.462		0
50	rs57326588	0.238	0.457		96
51	rs10034735	0.105	0	0	100
52	rs57497036	0.237	0.466		95.3
53	rs11931532	0.238	0.457	0.48	94.4
54	rs11944132	0.237	0.466	0.48	95
55	rs16892271	0.237	0.466		94
56	rs34102992	0.152	0.26		41.9
57	rs36097300	0.237	0.466		95.7
58	rs35016714	0.152	0.26		96.7
59	rs73123607	0.238	0.457		94.7
60	rs6844968	0.237	0.466	0.47	96.3
61	rs6828144	0.152	0.26	0.26	95.7
62	rs6845595	0.238	0.457		97.3
63	rs6845597	0.238	0.457		95
64	rs6449168	0.464	0.38		96.3
65	rs57987867	0.237	0.466		97.3
66	rs58978163	0.237	0.466		96
67	rs16892272	0.238	0.457	0.46	95.3
68	rs73123615	0.238	0.457		96.3
69	rs4292325	0.099	0.53	0.04	97.3
70	rs16892276	0.24	0.46	0.46	96.7
71	rs60913985	0.238	0.457		96.3
72	rs12651052	0.187	0.457	0.46	95
73	rs56381553	0.187	0.457		97.7
74	rs4616747	0.89	0		97
75	rs12645513	0.187	0.457	0.49	96.7
76	rs58335439	0.187	0.457		94.7
77	rs4588454	0.1	0.053	0.04	97.7
78	rs73123625	0.187	0.457		96.7
79	rs59919329	0.187	0.457		96.3
80	rs12645693	0.187	0.457	0.48	97
81	rs12646331	0.187	0.457	0.47	95.3
82	rs12643722	0.187	0.457		24.6
83	rs12640554	0.187	0.457		97.3

Table S1. *Cont.*

#	SNPs (dbSNP)	Minor Allele Frequencies			Success Rate (%)
		1000 Genomes *		HapMap **	
		Global	JPT	JPT	
84	rs34559912	0.414	0.38		95
85	rs12644354	0.187	0.457		94.4
86	rs12640596	0.187	0.457		94.4
87	rs112043166	0.187	0.457		99.3
88	rs4492001	0.098	0.053		65.4
89	rs4389574	0.416	0.38		95
90	rs111541714	0.187	0.457		21.9
91	rs73125993	0.187	0.457		90.7
92	rs9790535	0.187	0.457		93.7
93	rs9790554	0.187	0.457		95
94	rs9790670	0.187	0.457	0.48	96.7
95	rs16892283	0.187	0.457	0.48	94
96	rs16892287	0.001	0	0.46	94.7
97	rs16892289	0.186	0.457	0.48	94.7
98	rs10516290	0.186	0.457	0.46	95.7
99	rs10516291	0.186	0.457	0.47	96.3
100	rs16898413	0.186	0.457	0.45	97.3
101	rs4328911	0.186	0.457		95.7
102	rs4336219	0.186	0.457		97.3
103	rs4435745	0.186	0.457	0.46	94.4
104	rs4498133	0.186	0.457	0.48	93.4
105	rs1058212	0.186	0.47	0.48	98.3
106	rs4336220	0.186	0.457		95.7
107	rs4259058	0.117	0		98.7
108	rs4342183	0.186	0.457		95.3
109	rs955411	0.1	0.053		98
110	rs955410	0.208	0.457		94.7
111	rs11724635	0.408	0.38		97.3
112	rs4266290	0.404	0.38		95
113	rs4403048	0.404	0.38		0
114	rs4698412	0.408	0.38		96
115	rs73224670	0.1	0.053		97.3
116	rs4273468	0.309	0.404		96.7
117	rs4698413	0.408	0.38		96.3
118	rs4613561	0.46	0.38		95.7
119	rs4538475	0.309	0.404		96.3
120	rs11943501	0.098	0.053		97.7
121	rs11934811	0.188	0.337		97.7

SNP numbering in this study; * The 1000 Genomes Browser [1]; ** HapMap Genome Browser release #27 [2]; JPT, Japanese in Tokyo, Japan.

Table S2. Comparison of genotype and allele frequencies of previously reported Parkinson's disease-associated SNPs at Kanazawa University Hospital for ASDs.

	**Cases**	**Control**	**Odds Ratio (95% CI)**	***p***
		rs11931532		
Genotype	(*n* = 143)	(*n* = 138)		
G/G	52 (36.4%)	46 (33.33%)	Referent	
A/G	69 (46.9%)	70 (50.73%)	0.85 (0.50, 1.4)	0.5970
A/A	24 (16.8%)	22 (15.94%)	0.97 (0.48, 1.9)	1.0000
Allele	(*n* = 286)	(*n* = 276)		
G	171 (59.8%)	162 (58.7%)	Referent	
A	115 (40.2%)	114 (41.3%)	0.96 (0.68, 1.3)	0.7974
		rs12645693		
Genotype	(*n* = 143)	(*n* = 146)		
A/A	57 (39.9%)	65 (44.5%)	Referent	
A/G	63 (44.06%)	58 (39.7%)	1.2 (0.75, 2.1)	0.4424
G/G	23 (16.08%)	23 (15.8%)	1.1 (0.58, 2.1)	0.7317
Allele	(*n* = 288)	(*n* = 292)		
A	177 (61.5%)	188 (64.4%)	Referent	
G	109 (37.9%)	104 (35.6%)	1.1 (0.79, 1.6)	0.5471
		rs11724635		
Genotype	(*n* = 145)	(*n* = 145)		
G/G	66 (45.52%)	57 (39.3%)	Referent	
G/T	65 (44.83%)	69 (47.6%)	0.81 (0.50, 1.3)	0.4542
T/T	14 (9.66%)	19 (13.1%)	0.64 (0.29, 1.4)	0.3271
Allele	(*n* = 290)	(*n* = 290)		
G	197 (67.93%)	183 (63.1%)	Referent	
T	93 (32.07%)	107 (36.9%)	0.81 (0.57, 1.1)	0.2561
		rs4698412		
Genotype	(*n* = 145)	(*n* = 141)		
G/G	66 (45.52%)	54 (38.3%)	Referent	
A/G	65 (44.83%)	69 (48.9%)	0.77 (0.47, 1.3)	0.3168
A/A	14 (9.66%)	18 (12.8%)	0.64 (0.29, 1.4)	0.3201
Allele	(*n* = 290)	(*n* = 282)		
G	197 (67.93%)	177 (62.8%)	Referent	
A	93 (32.07%)	105 (37.2%)	0.80 (0.56, 1.1)	0.2185
		rs4273468		
Genotype	(*n* = 145)	(*n* = 143)		
T/T	59 (40.69%)	55 (38.5%)	Referent	
C/T	71 (48.97%)	71 (49.7&)	0.93 (0.57, 1.5)	0.8024
C/C	15 (10.34%)	17 (11.9%)	0.82 (0.37, 1.8)	0.6913
Allele	(*n* = 290)	(*n* = 286)		
T	192 (65.17%)	181 (63.3%)	Referent	
C	104 (34.83%)	105 (36.7%)	0.93 (0.67, 1.3)	0.7298

Table S2. *Cont.*

	Cases	Control	Odds Ratio (95% CI)	*p*
		rs4538475		
Genotype	(*n* = 144)	(*n* = 143)		
C/C	58 (40.28%)	55 (38.5%)	Referent	
C/T	65 (45.14%)	69 (48.3%)	0.89 (0.54, 1.5)	0.7024
T/T	21 (14.58%)	19 (13.3%)	1.0 (0.51, 2.2)	1.0000
Allele	(*n* = 289)	(*n* = 286)		
C	181 (62.63%)	179 (62.6%)	Referent	
T	107 (37.02%)	107 (37.4%)	0.99 (0.71, 1.4)	1.0000

CI, confidence interval; *p*-Values obtained by Fisher's exact test are given.

Table S3. The genotype distributions of the studied SNPs between cases and controls and their risk prediction for ASDs under three genetic models of inheritance.

	Cases	Control	Odds Ratio (95% CI)	*p*	Effect Size, w	Chi-Squared Power
rs4301112	(*n* = 145)	(*n* = 146)				
			Additive			
A/A	129 (88.9%)	144 (98.6%)	Referent			
G/G	2 (1.4%)	1 (0.7%)	2.23 (0.20, 24.9)	0.6054	0.193	0.6394
			Dominant			
A/A + A/G	143 (98.6%)	145 (99.3%)	Referent			
G/G	2 (1.4%)	1 (0.7%)	2.02 (0.18, 22.6)	0.6224	0.169	0.5285
			Recessive			
A/A	129 (88.9%)	144 (98.6%)	Referent			
A/G + G/G	16 (11.0%)	2 (1.4%)	8.9 (2.01, 39.6)	***0.0005***	0.525	1.0000
rs28532698	(*n* = 145)	(*n* = 143)				
			Additive			
A/A	129 (88.9%)	141 (98.6%)	Referent			
G/G	2 (1.4%)	1 (0.7%)	2.19 (0.20, 24.4)	0.6090	0.188	0.6199
			Dominant			
A/A + A/G	143 (98.6%)	142 (99.3%)	Referent			
G/G	2 (1.4%)	1 (0.7%)	2.00 (0.18, 22.0)	1.0000	0.166	0.5170
			Recessive			
A/A	129 (88.9%)	141 (98.6%)	Referent			
A/G + G/G	16 (11.0%)	2 (1.4%)	8.7 (2.0, 38.8)	***0.0009***	0.519	1.0000
rs10001565	(*n* = 145)	(*n* = 143)				
			Additive			
C/C	130 (89.7%)	141 (98.6%)	Referent			
T/T	1 (0.7%)	1 (0.7%)	1.08 (0.07, 17.5)	1.0000	0.019	0.0557

Table S3. *Cont.*

	Cases	Control	Odds Ratio	*p*	Effect Size, w	Chi-Squared Power
			Dominant			
C/C + C/T	144 (99.3%)	142 (99.3%)	Referent			
T/T	1 (0.7%)	1 (0.7%)	1.00 (0.06, 15.9)	1.0000	0.000	0.050
			Recessive			
A/A	130 (89.7%)	141 (98.6%)	Referent			
C/T + T/T	15 (10.3%)	2 (1.4%)	8.1 (1.83, 36.3)	***0.0018***	0.502	1.0000

CI, confidence interval; *p*-Values obtained by Fisher's exact test are given. Standard statistical package Stata 12, considering the Woolf approximation was used for this analysis. Significant *p*-values after multiple testing correction for effective total number of SNPs ($p < 0.002$) are written in bold and italicized. Chi-squared power calculation was done by statistical package R.

Table S4. *p*-Values for Hardy-Weinberg proportion tests.

	Pearson's Chi-Square Goodness-of-Fit Test		Likelihood Ratio Test	
	Cases	Controls	LRT (D)	LRT (R)
rs430112	0.0398	1.11E−15	0.0001	0.0870
rs28532698	0.0398	2.19E−15	0.0001	0.0876
rs10001565	0.3735	2.19E−15	0.0030	0.0993

LRT (D), likelihood ratio test of population HWE under dominant disease model; LRT (R), likelihood ratio test of population HWE under recessive disease model. Detailed data for LRT (D) and LRT (R) are given in Supplementary Tables S6 and S7, respectively.

Table S5. Likelihood ratio test of population Hardy-Weinberg equilibrium for rs4301112, rs28532698 and rs10001565 polymorphisms under dominant disease model.

Genotype Distribution (Cases/Controls)	Disease Model	*q*	α	β	γ	Deviance	*p*
				rs4301112			
AA (129/144)<br>AG (14/1)<br>GG (2/1)	Dominant model H_0	0.017	0.014	3.636	3.636	16.077	0.0003
	Dominant model H_a	0.009	0.014	8.064	8.064	1.391	0.2382
	Likelihood ratio test of H_0 versus H_a					14.686	0.0001
	Difference of deviance					14.686	
				rs28532698			
AA (129/141)<br>AG (14/1)<br>GG (2/1)	Dominant model H_0	0.017	0.014	3.562	3.562	15.96264	0.0003
	Dominant model H_a	0.009	0.014	7.914	7.914	1.391	0.2382
	Likelihood ratio test of H_0 versus H_a					14.572	0.0001
	Difference of deviance					14.572	

Table S5. *Cont.*

Genotype Distribution (Cases/Controls)	Disease Model	*q*	α	β	γ	Deviance	*p*
			rs10001565				
CC (130/141) CT (14/1) TT (1/1)	Dominant model H_0	0.014	0.014	4.111	4.111	10.976	0.0041
	Dominant model H_a	0.009	0.014	7.412	7.412	2.195	0.1385
	Likelihood ratio test of H_0 versus H_a					8.782	0.0030
	Difference of deviance					8.782	

p-Values obtained by Fisher's exact test are given. *q*, MAF in the population; α, The baseline disease penetrance in homozygotes of major alleles; β, The relative risk of disease for the heterozygotes in reference to homozygotes of major alleles; γ, The relative risk of disease for the homozygotes of minor alleles.

Table S6. Likelihood ratio test of population Hardy-Weinberg equilibrium for rs4301112, rs28532698 and rs10001565 polymorphisms under recessive disease model.

Genotype Distribution (Cases/Controls)	Disease Model	*q*	α	β	γ	Deviance	*p*
			rs4301112				
AA (129/144) AG (14/1) GG (2/1)	Recessive model H_0	0.031	0.015	1.0	13.237	17.185	0.0002
	Recessive model H_a	0.033	0.015	1.0	1.997	14.257	0.0002
	Likelihood ratio test of H_0 versus H_a					2.928	0.0870
	Difference of deviance					2.928	
			rs28532698				
AA (129/141) AG (14/1) GG (2/1)	Recessive model H_0	0.031	0.015	1.0	13.006	16.894	0.0002
	Recessive model H_a	0.033	0.015	1.0	1.957	13.977	0.0002
	Likelihood ratio test of H_0 versus H_a					2.918	0.0876
	Difference of deviance					2.918	
			rs10001565				
CC (130/141) CT (14/1) TT (1/1)	Recessive model H_0	0.030	0.015	1.0	6.750	16.596	0.0002
	Recessive model H_a	0.033	0.015	1.0	0.986	13.879	0.0002
	Likelihood ratio test of H_0 versus H_a					2.717	0.0993
	Difference of deviance					2.717	

p-Values obtained by Fisher's exact test are given. *q*, MAF in the population; α, The baseline disease penetrance in homozygotes of major alleles; β, The relative risk of disease for the heterozygotes in reference to homozygotes of major alleles; γ, The relative risk of disease for the homozygotes of minor alleles.

Table S7. Allele and genotype frequencies of SNPs (rs430112, rs28532698 and rs10001565) in unselected Japanese populations in public databases and *p*-values for Hardy-Weinberg proportion tests.

	Allele Frequencies		Genotype Frequencies		***p***
		rs4301112			
1000 Genomes	T	202 (97.1%)	T/T	98 (94.2%)	
:phase_3:JPT	C	6 (2.9%)	C/T	6 (5.8%)	0.762
			C/C	0 (0%)	(1.00)
		rs28532698			
1000 Genomes	T	202 (97.1%)	T/T	98 (94.2%)	
:phase_3:JPT	C	6 (2.9%)	C/T	6 (5.8%)	0.762
			C/C	0 (0%)	(1.00)
		rs10001565			
1000 Genomes	C	202 (97.1%)	C/C	98 (94.2%)	
:phase_3:JPT	T	6 (2.9%)	C/T	6 (5.8%)	0.762
			T/T	0 (0%)	(1.00)
HapMap-JPT	C	166 (96.5%)	C/C	80 (93.0%)	
ss13847282	T	6 (3.5%)	C/T	6 (7.0%)	0.737
			T/T	0 (0%)	(1.00)
Human Variation DB	C	385 (97.2%)	C/C	187 (94.4%)	
Study ID: 2_1	T	11 (2.8%)	C/T	11 (5.5%)	0.688
			T/T	0 (0%)	(1.00)
Human Variation DB	C	380 (97.9%)	C/C	186 (95.8%)	
Study ID: 10_1	T	8 (2.1%)	C/T	8 (4.1%)	0.769
			T/T	0 (0%)	(1.00)
Human Variation DB	C	220 (97.3%)	C/C	107 (94.6%)	
Study ID: 0_1	T	6 (2.7%)	C/T	6 (5.5%)	0.772
			T/T	0 (0%)	(1.00)

Hardy-Weinberg equilibrium in Japanese populations was tested using data from the Genome 1000 Project [1] and SNP Control Database [3]. JPT, Japanese in Tokyo. CI, confidence interval. *p*-Values for both Pearson's chi-square goodness-of-fit and Fisher's exact tests were calculated; those by Fisher's exact test are given in parentheses.

References

1. 1000 Genomes: A Deep Catalog of Human Genetic Variation. Available online: http://browser.1000genomes.org/index.html (accessed on 5 May 2015).
2. International HapMap Project. Available online: http://hapmap.ncbi.nlm.nih.gov/cgi-perl/gbrowse/hapmap27_B36/ (accessed on 5 May 2015).
3. The SNP Control Database. Available online: http://gwas.biosciencedbc.jp/snpdb/snp_top.php (accessed on 5 May 2015).
